# A dynamic nomogram for predicting diabetic macular edema in type 2 diabetes patients based on plasma cytokines

**DOI:** 10.18632/aging.202647

**Published:** 2021-03-03

**Authors:** Ning Zhang, Jing Ke, Dawei Zhang, Yuanyuan Zhang, Ying Fu, Bin Cao, Dong Zhao

**Affiliations:** 1 Center for Endocrine Metabolism and Immune Diseases, Beijing Luhe Hospital, Capital Medical University, Beijing 101149, China; 2 Beijing Key Laboratory of Diabetes Research and Care, Beijing 101149, China; 3 Department of Ophthalmology, Beijing Luhe Hospital, Capital Medical University, Beijing 101149, China

**Keywords:** plasma cytokines, diabetic macular edema, type 2 diabetes mellitus, dynamic nomogram

## Abstract

Objective: This study investigated changes of plasma cytokines and aimed to build a dynamic nomogram for diabetic macular edema (DME) in type 2 diabetes mellitus (T2DM).

Methods: In a pilot cohort, plasma samples were selected from 9 T2DM patients and 9 DME patients to screen for cytokine differences. The screening cytokines were then validated by enzyme-linked immunoassay in a cohort, which contained 100 DME (DME group) and 100 T2DM patients without DME (T2DM group). A dynamic nomogram for predicting DME was developed, based on the plasma cytokines.

Results: In the pilot cohort, 11 plasma cytokines were significantly increased in the DME group. In the validation cohort, platelet-derived growth factor (PDGF)-BB, tissue inhibitors of metalloproteinase (TIMP)-1, angiopoietin (ANG-1), and vascular endothelial cell growth factor receptor (VEGFR)-2 were confirmed to be significantly elevated in the DME group. The dynamic nomogram demonstrated good calibration and discrimination, with an area under the receiver operating characteristic curve (AUC) of 0.88. In the test set, sensitivity, specificity, and AUC were 73.3%, 80.0%, and 0.84, respectively.

Conclusion: Plasma cytokines were closely associated with DME. A novel dynamic monogram including ANG-1, PDGF-BB, TIMP-1, and VEGFR2 was a novel tool for predicting DME.

## INTRODUCTION

Diabetic macular edema (DME) is one of the most common microvascular complications in patients with type 2 diabetes mellitus (T2DM) and is a major cause of vision loss worldwide. The prevalence of DME depends on the type and duration of diabetes, and the incidence of DME is higher in individuals with type 1 diabetes than in those with T2DM. Approximately 27% of patients develop DME within 9 years of diabetes onset [1]. Optical coherence tomography (OCT) is an effective and sensitive imaging tool for detecting DME as long as no other causes of cystoid macular edema are present [2]. The fact that patients with macular edema may be asymptomatic and easy to be neglected provides strong support for screening to detect DME [3]. Although the OCT imaging-based features of DME are well-known, knowledge about its protein phenotype is limited.

Angiogenesis and inflammation response are key mechanisms of DME development [4]. ANG-1 expression in preretinal cells activates transmembrane tyrosine kinase Tie2, which is highly expressed in the endothelium and promotes endothelial intercellular links through multiple pathways, thereby reducing endothelial cell permeability [5–8]. MMP-9/TIMP-1 are critical for maintaining the integrity and impermeability of the blood–retina barrier, and retinal damage occurs when the MMP-9/TIMP-1 ratio is out of balance [9]. PDGF-BB can upregulate VEGF expression and promote angiogenesis after binding to the receptor on the surface of human retinal vascular endothelial cells (hRVECs) [10]. However, data on the association between plasma cytokines and DME is limited.

To explore the potential role of plasma angiogenesis and inflammation cytokines in DME, we investigated the levels of vascular and inflammatory cytokines by protein-chip screening and enzyme-linked immunosorbent assays (ELISAs). A dynamic nomogram for predicting the risk of DME in patients with T2DM was developed and was based on the DME-associated plasma cytokines.

## RESULTS

### Study subjects

The general characteristics of the study subjects are shown in Table 1. For plasma protein profiling, 9 DME patients and 9 T2DM patients were selected as a pilot cohort. For validation, 100 DME patients and 100 T2DM patients were enrolled. No significant differences in age, body mass index, duration of diabetes, fasting plasma glucose, hemoglobin (Hb)A1c, fasting C-peptide, 2-h post prandial C-peptide, and gender were found between the two groups in either the pilot or the validation cohort. There were also no between-group differences in diabetes-related complications.

**Table 1:** Comparisons of clinical characteristics of the study cohort.

**Clinical characteristics**	**Pilot cohort**				**Validation cohort**		
	**T2DM (n=9)** **(Mean ± SD)**	**DME (n=9)** **(Mean± SD)**	**p**		**T2DM (n=100)** **(Mean ± SD)**	**DME (n=100)** **(Mean ± SD)**	**p**
**Age** **(years)**	59.44±8.82	61.00±10.48	0.738		62.41±12.43	59.76±11.86	0.141
**BMI** **(Kg/m^2^)**	26.17±1.74	26.36±3.89	0.895		25.48±3.82	25.80±3.60	0.573
**Duration of diabetes (years)**	6.56±9.65	8.78±6.85	0.682		7.40±8.63	7.48±7.31	0.951
**Fasting plasma glucose (mmol/L)**	6.58±2.38	6.89±1.68	0.758		8.73 ±3.26	8.26 ±4.18	0.437
**HbA1c** **(%)**	10.2±2.34	9.66±1.79	0.573		9.90 ±2.02	9.52 ±2.55	0.269
**Fasting C peptide (mIU/L)**	1.47±0.66	1.63±1.06	0.697		1.73 ±1.18	1.79 ±1.19	0.737
**2-h post prandial C-peptide (mIU/L)**	5.80±4.30	3.68±2.01	0.205		3.96 ± 3.28	4.60 ± 2.76	0.183
**Triglyceride** **(mmol/L)**	2.09±1.44	2.05±1.91	0.963		1.96 ± 2.32	1.65 ± 0.95	0.279
**Total cholesterol (mmol/L)**	5.04±2.69	4.24±1.50	0.481		4.37 ± 1.39	4.38 ± 1.03	0.933
**Low-density lipoprotein** **(mmol/L)**	3.27±1.91	2.48±1.14	0.334		2.77 ± 1.07	2.85 ± 0.84	0.606
**Gender,** **male (%)**	5(55.6%)	4 (44.4%)	1.000		53 (53.0%)	46 (46.0%)	0.396
**Hypertension,** **number (%)**	4 (44.4%)	6(66.7%)	1.000		50 (50.0%)	44 (44.0%)	0.479
*Diabetic nephropathy, **number (%)**	3 (33.3%)	5 (55.6%)	0.635		40 (42.6%)	29 (31.5%)	0.160
**Diabetic peripheral neuropathy,** **number (%)**	0 (0%)	0 (0%)	1.000		1 (1.0%)	1 (1.0%)	1.000

*, 14 missing data of diabetic nephropathy in validation cohort.

### Multiple cytokine alterations in DME plasma

**Figure 1:**
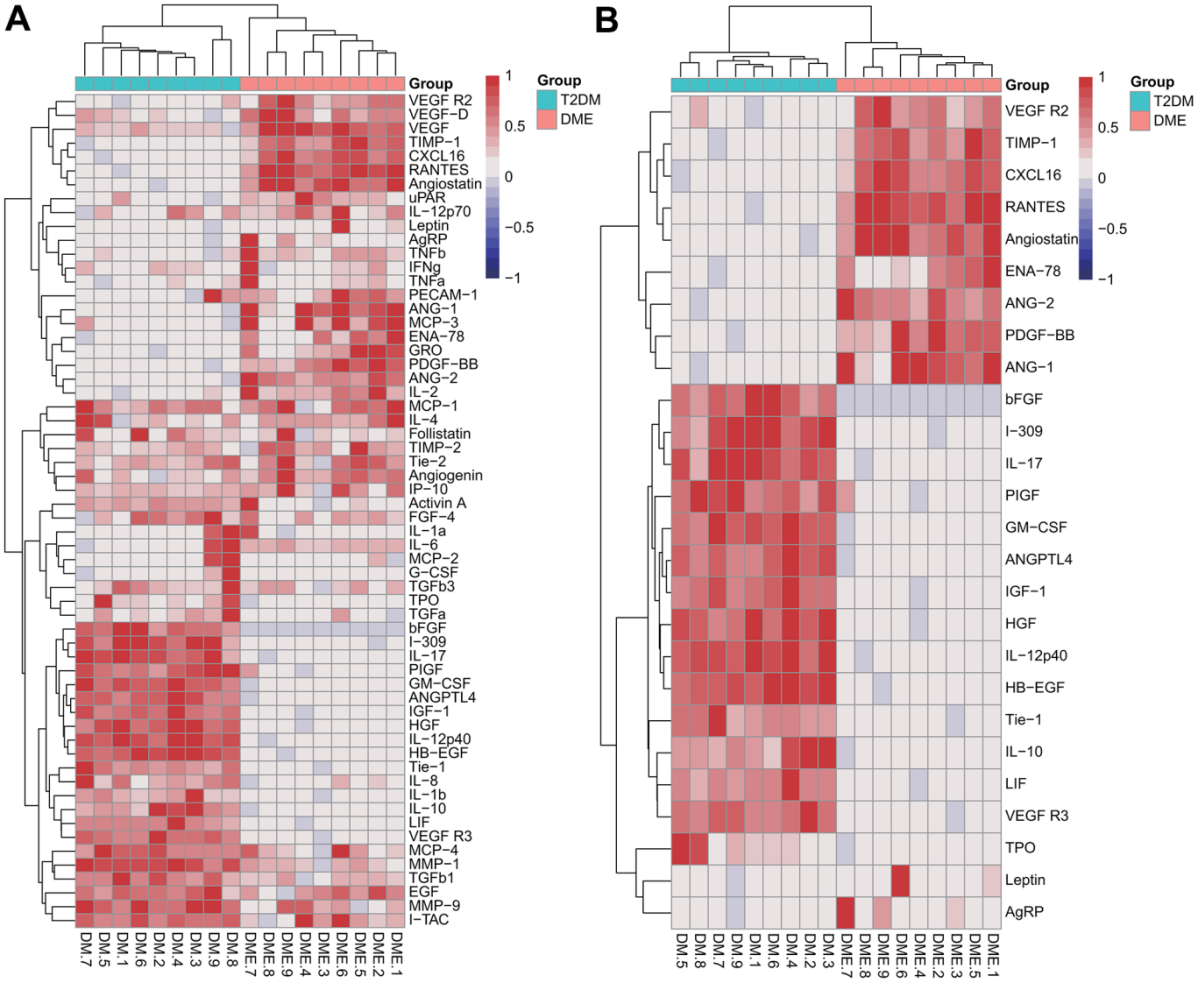
**Semi-quantitative results of cytokine analysis in the pilot cohort.** Heatmaps of the relative changes of 60 plasma cytokines (**A**) and 26 cytokines with a fold change > 4 or < 0.25 (**B**).

To profile plasma cytokines, blinded screening by protein microarray analysis was performed, and semi-quantitative results were obtained for 60 plasma proteins. The relative changes of plasma cytokines are shown in the heatmap in Figure 1A. Compared with the T2DM group, the levels of 15 cytokines were significantly lower in the DME group and 11 were significant higher, with more than a four-fold change (adjusted *p* < 0.05, Figure 1B). The increases in 5 plasma proteins, PDGF-BB, TIMP-1, ANG-1, CXCL16, and VEGFR2 in the DME group were greater than six-fold. Principal component analysis (PCA) found a clear distinction between the two groups, suggesting that these 5 plasma proteins might be helpful to distinguish T2DM patients with and without DME (Supplementary Figure 1).

### ELISA validation of five markedly increased cytokines

**Figure 2:**
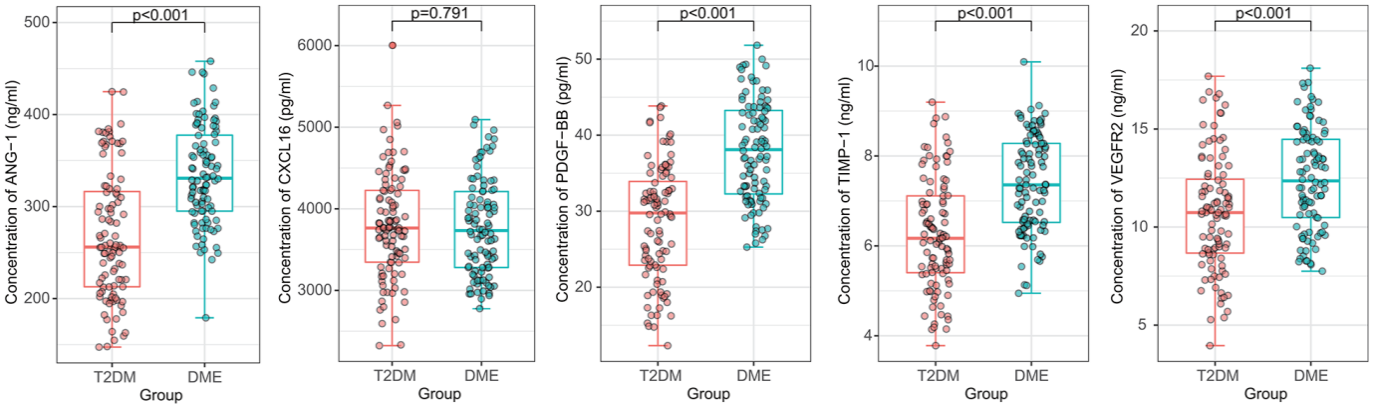
**Comparison of the plasma concentrations of ANG-1, CXCL16, PDGF-BB, TIMP-1, and VEGFR2 in the validation cohort.** ANG-1, PDGF-BB, TIMP-1 and VEGFR2 concentrations were significantly increased in DME samples. The between-group difference of CXCL16 concentration was not significant.

To further determine whether ANG-1, CXCL16, PDGF-BB, TIMP-1, and VEGFR2 levels were significantly increased in DME, 100 DME patients and 100 T2DM patients were recruited to validate the array data using ELISA. As shown in Figure 2, the mean levels of ANG-1 (334.62 ng/ml), PDGF-BB (37.86 pg/ml), TIMP-1 (7.39 ng/ml), and VEGFR2 (12.47 ng/ml) in the DME group were significantly higher than those in the T2DM group (268.94 ng/ml, 28.60 pg/ml, 6.27 ng/ml, and 10.71 ng/ml), respectively (*p* < 0.001). Between-group differences in CXCL16 (3776.43 pg/ml vs. 3794.39 pg/ml) were not significant (*p* = 0.791).

### Correlation of cytokine levels and clinical characteristics in DME group

**Figure 3:**
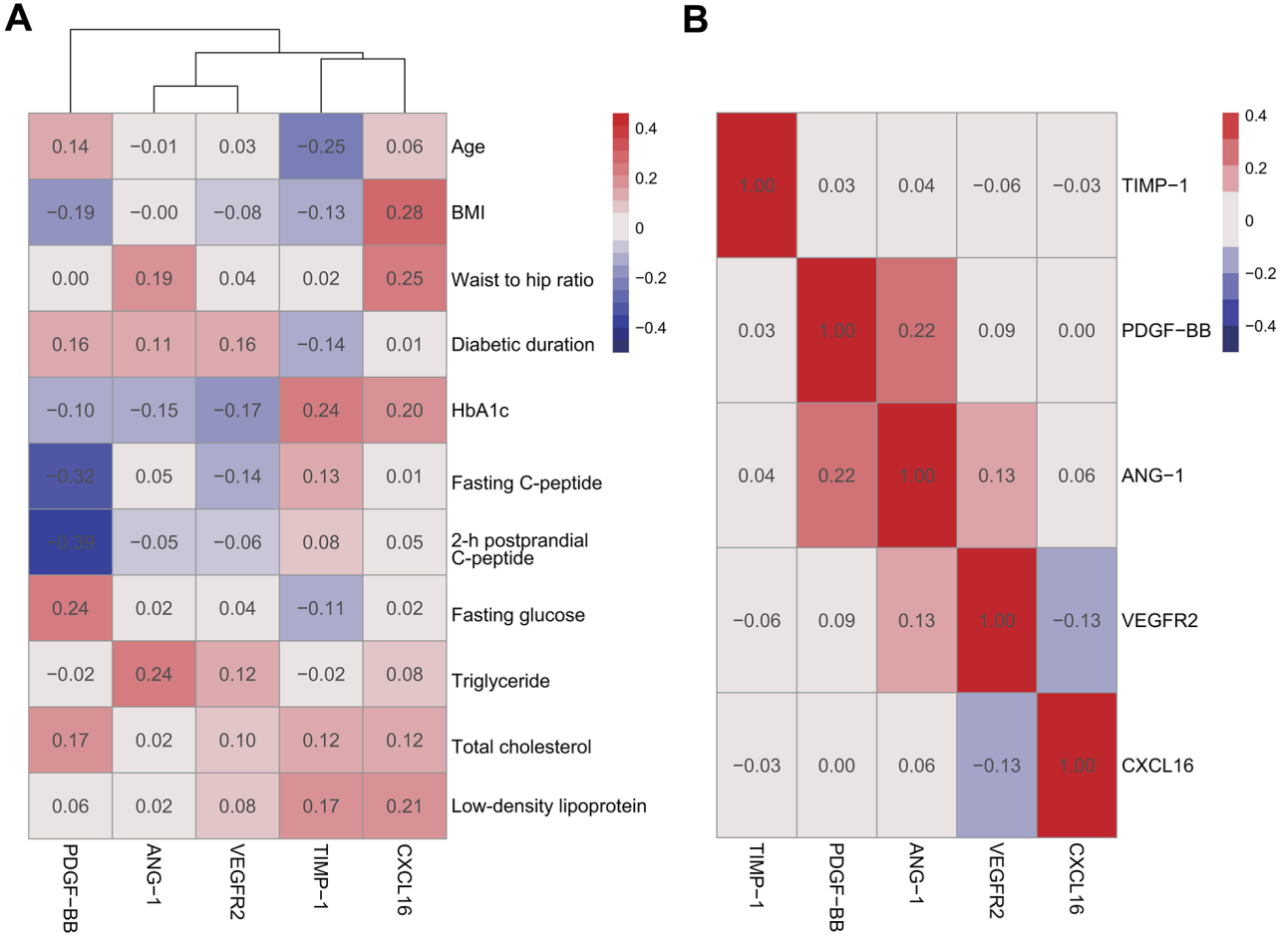
**Pearson correlation analysis of DME group.** There were no significant positive correlations between plasma cytokines and clinical characteristics (**A**) among plasma cytokines (**B**).

As shown in the heatmap (Figure 3A), Pearson correlation analysis of the relationships of cytokine levels and clinical features found no significant positive correlations between clinical characteristics, including age, body mass index, waist-to-hip ratio, diabetes duration, fasting blood glucose, HbA1c, fasting C-peptide, 2-h postprandial C-peptide, and the 5 plasma cytokines, including ANG-1, PDGF-BB, TIMP-1, VEGFR2 and CXCL16 (*r* < 0.3). Focusing on the inner relationship of cytokines, there was also no obvious correlation among these 5 cytokines (*r* < 0.3, Figure 3B).

### PCA for cytokine selection

**Figure 4:**
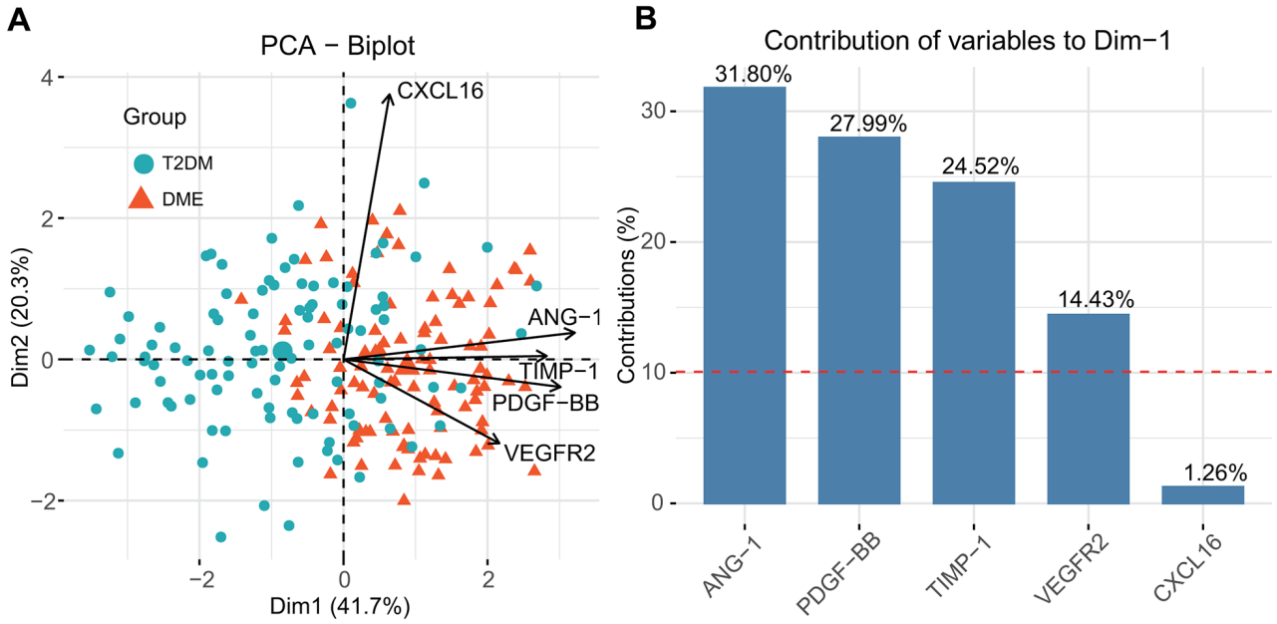
Principal component analysis (PCA) for feature selection and computation of the relative contribution of each cytokine to distinguishing DME and T2DM (**A**) and the contribution of each cytokines to the first principal component (**B**).

PCA was performed to determine the relative contribution of each cytokine to the ability to distinguish DME- and T2DM-group patients. The first and second principal components of the PCA plot (Dim1 and Dim2) accounted for 41.7%, and 20.3% of the variation, respectively, in the dataset. The projection of samples in the PCA revealed relatively little overlapping of areas. CXCL16 contributed more to the second than to the first principal component. ANG-1, PDGF-BB, TIMP-1, and VEGFR2 contributed more to the first principal component (Figure 4A). As shown in Figure 4B, the percentage contributions of cytokines to first principal component were ANG-1 (31.80%), PDGF-BB (27.99%), TIMP-1 (24.52%), VEGFR2 (14.43%), and CXCL16 (1.26%). According to contribution percent, ANG-1, PDGF-BB, TIMP-1, and VEGFR2 contribute more to distinguish T2DM patients with and without DME. To reduce overfitting, ANG-1, PDGF-BB, TIMP-1, and VEGFR2 were included in a logistic regression analysis.

### Development and validation of logistic regression and nomogram development

**Figure 5:**
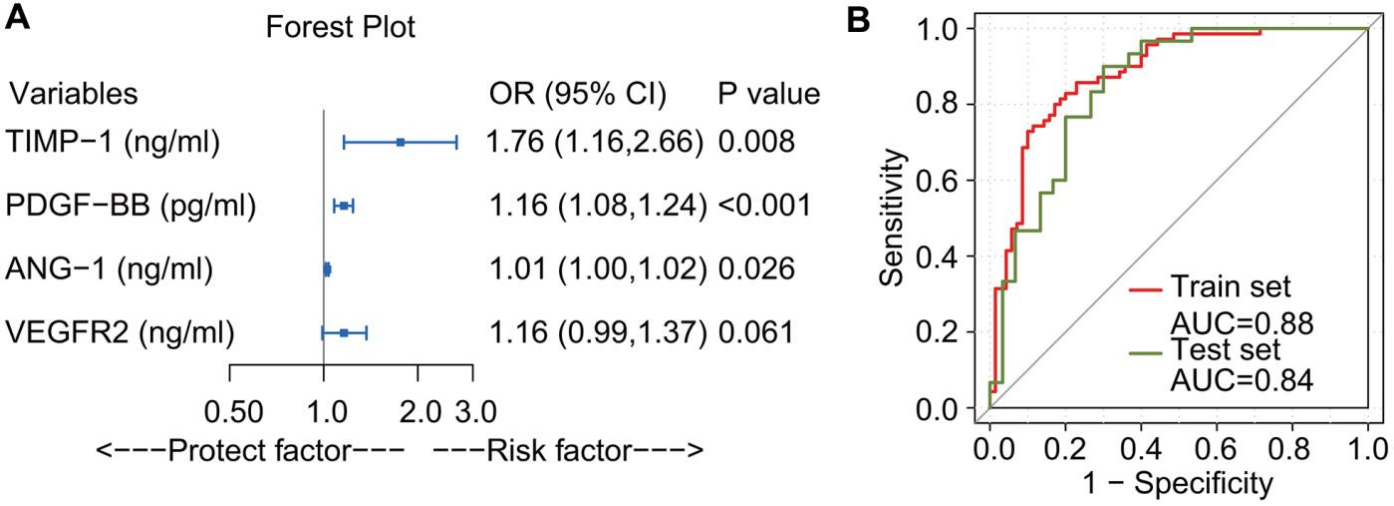
**Forest plot and receiver operating characteristic curve of logistic regression.** ANG-1, PDGF-BB, and TIMP-1 were identified as risk factors. VEGFR2 did not reach statistical significance, (*p*-value slightly larger than 0.05). Because the Akaike Information Criterion increased from 129.65 to 131.32 when removing VEGFR2 from the model, it was retained (**A**). Receiver operating characteristic curve for the prediction model. The area under the receiver operating characteristic curve of the training set (red line) and the test set (olive drab line) were 0.88 and 0.84, respectively (**B**).

Multivariate logistic regression was used to develop a model to predict DME. As shown in Figure 5A, ANG-1 (odds ratio [OR] = 1.01, 95% confidence interval [CI]: 1.00–1.02; *p* = 0.026], PDGF-BB (OR = 1.16, 95% CI: 1.08–1.24, p<0.001), and TIMP-1 (OR = 1.76, 95% CI: 1.16–2.66, *p* = 0.008) were identified as significant risk factors. VEGFR (*p* =0.061) did not reach statistical significance. However, as Akaike information criterion (AIC) increased from 129.65 to 131.32 when removing VEGFR2 from the model, it was retained in the analysis. Figure 5B shows that the logistic regression model performed well in the training dataset, with an area under the receiver operating characteristic curve (AUC) of 0.88. In the test set, the sensitivity was 73.3%, specificity was 80.0% and the AUC was 0.84.

**Figure 6:**
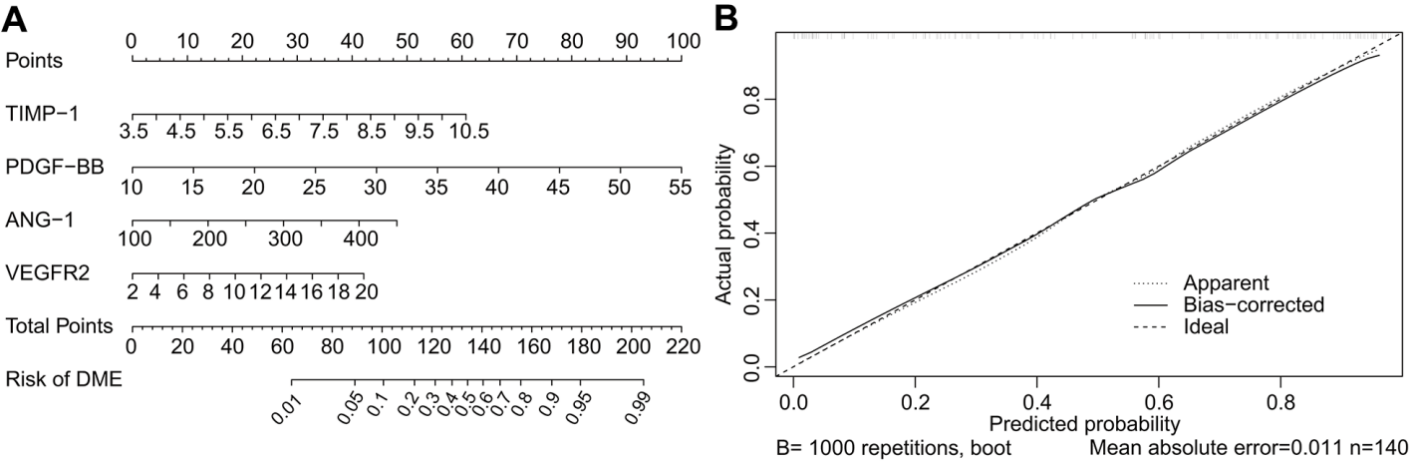
**Nomogram and calibration curve for predicting DME.** The DME-risk nomogram includes ANG-1, PDGF-BB, TIMP-1, and VEGFR2 as predictors (**A**). The calibration curve for predicting DME (**B**). The nomogram-predicted probability of DME is plotted on the *X*-axis and the actual probability is plotted on the *Y*-axis. Calibration curves demonstrated satisfactory consistency between the predicted risk and the actual probability.

Based on the results of multivariate logistic regression, a nomogram including ANG-1, PDGF-BB, TIMP-1, and VEGFR2 was constructed for DME risk prediction (Figure 6A). The point score of each variable was based on its contribution to the model and the point total corresponds to the risk and predicted likelihood of DME. Calibration curves demonstrated good consistency between the predicted risk and the actual probability (Figure 6B). The apparent curve confirmed the good prediction capability of the nomogram.

### Clinical usefulness of the nomogram

**Figure 7:**
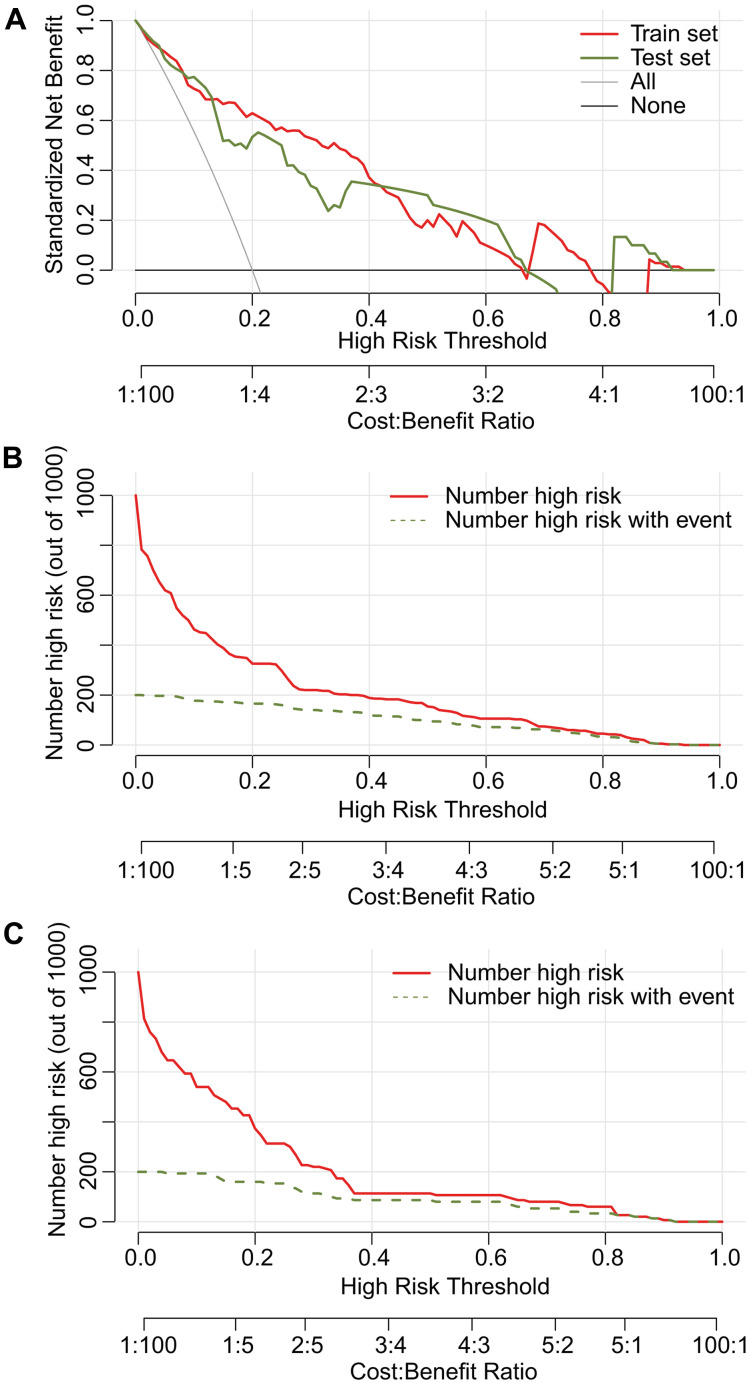
**Decision curve analysis (DCA) and clinical impact plot of the nomograms for DME in both the training and test sets.** The net benefit is greater than that achieved with an all-or-none patient intervention scheme if the threshold probability is less than 60% (**A**). Predictive high-risk estimates were similar to the actual number of event cases when the threshold probability was > 0.3 in both the training (**B**) and test set (**C**).

The decision curve demonstrated that in both the training and test datasets, the net benefit was greater than with an all-or-none patient intervention scheme if the threshold probability was less than 60%, which supports use of the nomogram in clinical practice (Figure 7A). A clinical impact curve was plotted to predict the number of high-risk patients in a population of 1000. In both the training and test datasets, the predicted high-risk number was close to the actual number of event cases when the threshold probability was greater than 0.3, and the cost-benefit ratio was close to 2:5 (Figures 7B, 7C). The dynamic nomogram is accessible online as user-friendly digital interface (https://doctorcao.shinyapps.io/DynNomapp/).

## DISCUSSION

DME is a type of retinal vascular disease. Angiogenic and inflammatory processes mediate the genesis of DME, and it is important to define which processes are enhanced or decreased in clinical practice. In the pilot cohort, we analyzed the expression changes of 60 angiogenic and inflammatory factors in DME by protein-chip screening. Eleven cytokines, including PDGF-BB, TIMP-1, ANG-1, and VEGFR2 were significantly increased in DME compared with those T2DM group. We expanded the sample size in the validation cohort and obtained consistent results that were used to develop a dynamic online nomogram for DME prediction. The user-friendly digital interface makes it easier to predict risk and to make better clinical decisions.

In this study, we highlighted the significance of increased expression of inflammatory and angiogenic factors in DME. Studies found that cytokines were elevated or decreased in the DME group and were closely related to the pathogenesis of vascular permeability and inflammation during the development of DME [11–13]. Our study confirmed that ANG-1, VEGFR2, TIMP1, and PDGF-BB, which were generally correlated with inflammatory process, were increased in DME. However, the pathological mechanisms of these four plasma cytokines during the progression of DME is unclear, and further studies are needed.

In this study, the characteristics of elevated vascular inflammatory factors in DME were used to establish a prediction model for DME. Previous clinical studies have reported elevated VEGF, MCP1, IL-6, and ICAM-1 were detected in vitreous fluids of DME patients, which is consistent with our results [14–22]. There are few published studies on the joint utilization of cytokines to predict DME. In the investigation of the pathogenesis of DME, we screened 60 angiogenic and inflammatory cytokines and finally selected four plasma cytokines for joint modeling.

The use of OCT-imaging biomarkers for evaluating therapeutic response has been described. Ceravolo et al. [23] demonstrated that the number of hyper-reflective spots (HRSs) and serous detachment of the neuroepithelium were specific noninvasive biomarkers for monitoring treatment response. Vujosevic et al. [24] reported that HRS number, extent of disorganization of the inner retinal layers, central macular thickness, and the cyst area of the deep capillary plexus were retinal biomarkers that may help in evaluating treatment response. Few studies have focused on the use of plasma proteins in the diagnosis of DME. In this study, we confirmed that plasma cytokines not only help to evaluate the risk of DME with good sensitivity and specificity, but may also provide new insights into the pathological mechanism of DME. The results have clinical significance for risk assessment and diagnosis when the use of OCT imaging is limited or not universally available.

We did not observe positive correlations between cytokines and indexes of glucose metabolism such as HbA1c or C-peptide in the DME group. The possible cause was that the study subjects were all hospital inpatients in the department of endocrinology for improved glucose management and control, which may have eliminated the effects of glucose level on the results. The association of cytokines with the course of disease or diabetic complications needs further investigation.

There were some limitations. First, we included about 200 subjects for model development and verification. Although the results were clear, there was no external cohort to validate the performance of the model. A large prospective study is needed for further validation. Second, coronary heart disease (CHD) may also result in abnormal expression of plasma proteins. However, the diagnosis of CHD was based on a history of disease, and as CHD in diabetes patients may be asymptomatic, a few CHD patients may have been included in this study.

In conclusion, plasma ANG-1, TIMP-1, PDGF-BB, and VEGFR2 were increased and were comprehensive predicators of DME when included in a dynamic nomogram. The nomogram needs further confirmation in large populations.

## MATERIALS AND METHODS

### Subjects

The study received ethical approval from the competent Institutional Review Boards of Beijing Luhe Hospital. All procedures complied with the ethical principles of Helsinki Declaration for studies of human subjects. The study was registered on clinicaltrials.gov (NCT03811470).

Patients were recruited at the Center for Endocrine Metabolism and Immune Diseases of Beijing Luhe Hospital, Capital Medical University, Beijing, China from January 2017 to December 2018. All patients participated in the program of the National Metabolic Management Center (MMC) [25]. 9 T2DM patients and 9 DME patients were recruited for a pilot study in which a protein antibody array was used to screen aberrantly expressed plasma protein. 100 DME patients (DME group) and 100 T2DM patients without DME or diabetic retinopathy (T2DM group) were recruited for a validation study using ELISA kits to verify the aberrantly expressed plasma proteins that were identified in the pilot study. T2DM patients with and without DME were eligible for inclusion. Patients with T1DM or other type of DM, with other types of retinopathy, with a history of any previous intravitreal injection or any other treatment for DME, or a history of cardiovascular diseases or stroke were excluded. The diagnosis of DME was determined by OCT [26], and confirmed by two senior ophthalmologists.

### Plasma sample collection

For the antibody arrays and ELISA, blood samples were collected at our center with ethylenediaminetetraacetate as the anticoagulant. The plasma was isolated by standard blood processing and the aliquots were frozen and stored at −80° C, avoiding freeze-thaw cycles.

### Cytokine antibody array screening

Cytokines were assayed in duplicate with RayBio R Human Cytokine Antibody Array (RayBiotech G-Series Human Angiogenesis Array 2 and G-Series Human Angiogenesis Array 3, RayBiotech). Spiking and recovery tests were performed to ensure a linear concentration response. As determined by densitometry, the interarray coefficient of variation of spot signal intensities was 20%. Cytokine results were presented as a heatmap using the “pheatmap” package.

### ELISA validation

Plasma PDGF-BB, TIMP-1, ANG-1, CXCL16, VEGFR2 levels were determined with human ELISA kits (Mlbio) following the manufacturer’s instructions. The intra-assay coefficient of variation was 20%, and the intra-assay coefficient of variation was 12%. No significant cross reactivity or interference was observed.

### Development of a dynamic nomogram

The patients in the validation cohort were randomly divided into training and test sets, which included 70% and 30% of the data, respectively. The cytokines most predictive of DME were selected by PCA using the data included in the training cohort. Multivariate logistic regression identified the cytokines significantly associated with DME development and they were used to construct a nomogram presenting a specific system for calculating the risk of DME. The performance of nomogram was evaluated by sensitivity, specificity, discrimination, and calibration. Discrimination, which is the predictive accuracy to distinguish patients with DME from those without DME, was measured by the receiver operating characteristic (AUC). A calibration curve, which reflected the consistency between the predicted probability and the actual probability, was plotted using 1000 bootstrap resamples. Decision curve analysis (DCA) was used to assess clinical usefulness of the nomogram. For access to risk estimation, a dynamic nomogram having a user-friendly digital interface was created online using the DynNom package [27].

### Statistical analysis

All data analysis and visualization were performed with in R software, version 3.6.3 (The R Project for Statistical Computing). Significance was evaluated by *t*-tests for normally distributed data; otherwise, the nonparametric Mann-Whitney test was used to analyze the data. Chi-square tests were performed for categorical variables. Pearson’s correlation analysis was performed to assess the relationships of the plasma cytokines. In all cases, *p*-values < 0.05 were considered statistically significant.

### Ethical approval

The study received ethical approval from the competent Institutional Review Boards of Beijing Luhe Hospital.

## Supplementary Material

Supplementary Figures
