# Supplementary material for: A dynamic nomogram for predicting diabetic macular edema in type 2 diabetes patients based on plasma cytokines
Source: Aging (Albany NY). 2021 Mar 3;13(6):8369–79. doi: 10.18632/aging.202647 (PMC8034887; doi:10.18632/aging.202647)
Supplement: Supplementary Figures [file aging-13-202647-s001.pdf]

SUPPLEMENTARY FIGURE

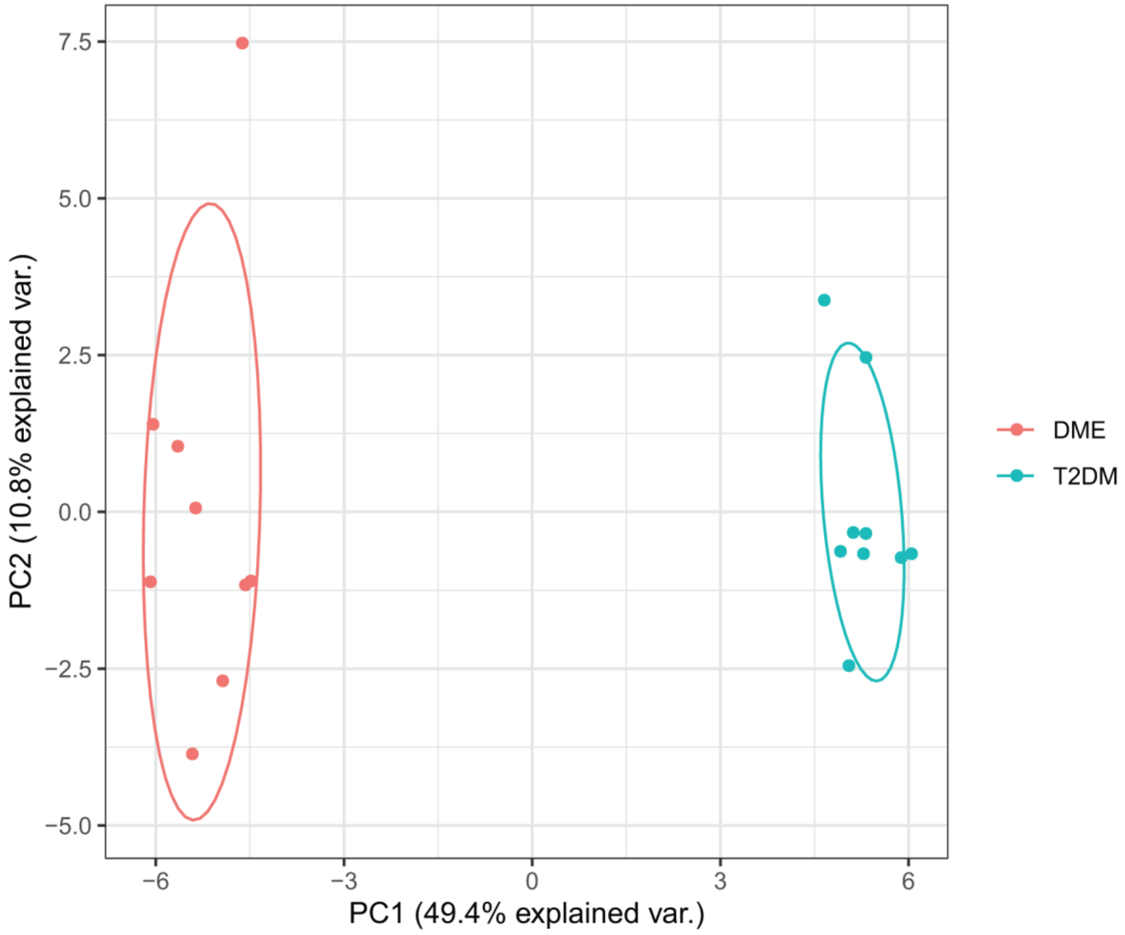

Supplementary Figure 1. Principle component analysis for the pilot cohort. There is a clear separation between the two groups.
